# Evaluation of *in vivo* bond strength and skin irritation test for new skin adhesive

**DOI:** 10.1016/j.jobcr.2023.10.001

**Published:** 2023-10-19

**Authors:** Paweena Kongkon, Wiwat Pichayakorn, Sasiwimol Sanohkan

**Affiliations:** a Department of Prosthetic Dentistry, Faculty of Dentistry, Prince of Songkla University, Songkhla, 90112, Thailand; b Department of Pharmaceutical Technology, Faculty of Pharmaceutical Sciences, Prince of Songkla University, Songkhla, 90112, Thailand

**Keywords:** Deproteinized natural rubber latex adhesive, Silicone prostheses, Maxillofacial prostheses, Skin adhesive

## Abstract

**Objectives:**

This study developed a new skin-deproteinized natural rubber latex (DNRL) silicone adhesive for adhering to silicone prostheses and compared the properties with a commercial Daro-Hydrobond adhesive.

**Materials and methods:**

The new DNRL skin adhesive formulation was made from non-vulcanized natural rubber-based adhesives consisting of DNRL, 20% polyvinyl alcohol, cumarone resin, 2% methylcellulose, and Wingstay L. The peel bond strength of the adhesives was tested using a 90-degree peel test. Biocompatibility was accessed using i*n vitro* keratinocyte cell viability. Animals (rabbits) and humans were tested for skin irritation tests. Results were analyzed using SPSS Version 24 and compared between the two adhesives.

**Results:**

The peel bond strength of the new DNRL skin adhesive was 103.61 ± 23.18 N/m whereas that of the Daro-hydrobond adhesive was 131.52 ± 21.72 N/m. There was no significant difference (*p>*0.05) between the peel bond strengths of the two test adhesives. Cell proliferation under the DNRL skin adhesive-soaked medium showed higher cell viability than the positive control (*p<*0.05). The DNRL skin adhesive produced moderate erythema and edema on rabbit skins, however, the skin lesions recovered within 14 days. Two volunteers showed mild irritation at the first hour of the contact which was reduced within an hour without any therapy. The patient satisfaction with the DNRL skin adhesive ranged from slightly satisfied to completely satisfied.

**Conclusion:**

The new DNRL skin adhesive showed comparable peel bond strength and patient satisfaction to those of commercial adhesives. The adhesive was biocompatible and can be used carefully.

## Highlights

• Skin adhesives are a good option for securing the extraoral prostheses to the skin.
• The new DNRL skin adhesive showed comparable peel bond strength.
• There was good patient satisfaction for the DNRL skin adhesive.
• The adhesive was biocompatible and can be used carefully.

## Graphical abstract

**Figure:**
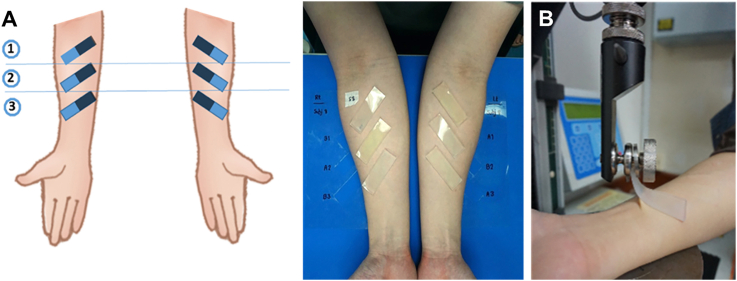

## 1 Introduction

Maxillofacial prosthetics deals with the rehabilitation and reconstruction of patients with facial deformities resulting from cancer, trauma, disease, or congenital defects. Silicone maxillofacial prostheses are of choice due to their superior physical, mechanical, and biological properties.^1^^,^^2^ Mechanical, anatomical, adhesive, or craniofacial implants are all viable options for retaining a maxillofacial prosthesis in position.^3^, ^4^, ^5^ Skin adhesives are a good option for securing the extraoral prostheses to the skin. After removing the prosthesis, the skin adhesive should be able to be removed completely. In addition to being non-irritating and non-allergic to the skin, it should be easily manipulated by the patient.

Two types of adhesives are used in maxillofacial prosthetics: silicone-based adhesives and water-based adhesives. The silicone-based adhesives were developed decades ago and have limitations, i.e. ethyl acetate causes significant skin irritation.^6^ Water-based adhesives frequently lack sufficient adhesion and produce a visible line between the prosthesis and the skin^7^,^8^. There are commercial adhesives available but are expensive. Polymer blends of deproteinized natural rubber latex (DNRL) have been used for medical applications, i.e. transdermal drug delivery, peel-off masks, and cosmetic pore packs.^9^, ^10^, ^11^, ^12^ It showed that the novel DNRL adhesive did not differ significantly from the commercial adhesive in terms of its physical properties and T-peel bond strength. The DNRL adhesive needs to be stored at 4 °C for six months to exhibit physical stability.^13^

In this study, we aimed to develop a new skin-deproteinized natural rubber latex (DNRL) silicone adhesive for adhering maxillofacial silicone prostheses and compared the properties with a commercial adhesive (Daro-Hydrobond adhesive). We tested biocompatibility, skin irritation, a 90-degree peel, and patient satisfaction for the new DNRL skin adhesive.

## 2 Materials and Methods

### 2.1 Materials

Table 1 shows the materials and chemicals used in this study and all chemicals were analytical grade. DNRL is used as a major component. Polyvinyl alcohol (PVA); Mw 67,000; was used to create polymers that are combined with DNRL. Coumarone indene resin was used as tackifier. Daro-Hydrobond adhesive, as a commercial adhesive was used as a positive control. Silicone strips were prepared from MDX4-4210® silicone elastomers.

**Table 1:** Materials used to prepare new skin adhesive in this study.

Material	Function	Manufacturer
Polyvinylalcohol (PVA)	Adhesive polymer	Sigma-aldrich, USA
Coumarone indene resin	Tackifier resin	Vega ball, Thailand
Methyl cellulose	Thickener	LOBA Chemie, India
Wing stay L	Antioxidant	LOBA Chemie, India
Daro-Hydrobond adhesive	Commercial adhesive	Factor II, Ariz, USA
MDX4-4210 ®	Silicone elastomer	Dow Corning Corp, Midland, MI, USA

## 3 Methods

### 3.1 Experimental adhesive preparation

Fresh NRL was deproteinized with the alcalase enzyme and processed using the centrifugation method to obtain DNRL as mentioned by Pichayakorn et al.^9^ The new DNRL skin adhesive was developed from non-vulcanized natural rubber-based adhesives. The adhesive formulations are shown in Table 2. The 15 phr of 20% PVA, 40 phr of 50% cumarone resin, 1 phr of 2% MC, and 1 phr of 50% Wingstay L were included. After the preparation, the new DNRL skin adhesive was stored at 4 °C. Then new DNRL skin adhesive and Daro-Hydrobond adhesives were tested for biocompatibility, irritation on the rabbit and human skin, and peel bond strength.

**Table 2:** Formulations of the new skin adhesive.

Components	Function	Manufacturer	Formulation (phr)	Ingredient percentage
60% DNRL	Main component	Processed	100	64
20% PVA	Adhesive polymer	Sigma-aldrich, USA	15	9.5
50% Coumarone resin	Tackifier resin	Vega ball, Thailand	40	25.5
2% MC	Thickener	LOBA Chemie, India	1	0.5
50% Wingstay L.	Antioxidant	LOBA Chemie, India	1	0.5

### 3.2 Biocompatibility testing (Cell viability study)

The cell viability tests were used to study the biocompatibility of the DNRL adhesive. The silicone MDX4-4210 was mixed (base/catalyst ratio 10:1). Silicone discs were fabricated (14 mm × 1.2 mm thickness) using a gypsum mold. The elastomer was cured at room temperature for 72 h. The silicone specimens were divided into three groups, as follows: silicone disc with Daro adhesive (positive control); blank pure silicone disc (negative control); and silicone disc with new DNRL skin adhesive test. Before testing, the silicone specimens were sterilized using gaseous sterilization at 55 °C for 4 h.

For cell viability, human immortalized non-tumorigenic keratinocyte cell line HaCaT (Caucasian; 62 years; Male skin; CLS Cell Lines Service, Eppelheim, Germany) following the ISO 10993-5.^14^ The cells were grown in T-25 flasks and were sub-cultured three times a week at 37 °C, in an atmosphere of 5% CO_2_ in air and 100% relative humidity, and the third passage was used. The cell culture medium consisted of DMEM with 10% (v/v) FBS and 0.1% antibiotic/antimycotic solution.

Either DNRL skin adhesive or Daro adhesive was applied on silicone discs and dried until the adhesive turned clear. Then, the discs were incubated in a 200 μL culture medium at 37 °C for 24 h. The ratio of the disc sample's surface area to the extraction volume was 3 cm^2^/mL. After the incubation, the extracts were filtered through 0.22 μm cellulose acetate filters to evaluate cytotoxicity. The multi-well plates were incubated at 37 °C with 5% CO_2_ in the air for 24 h. Then the culture medium was removed from each well and equal volumes (200 μL) of the extracts were added to the wells. In control wells, 200 μL medium was added. Then, 96-well cluster cell culture plates were incubated at 37 °C for 24, 48, and 72 h. After that, the test extracts were removed and replaced with 100 μL of PBS for washing the cell. Following the removal of the fetal bovine serum (PBS), the 3-(4,5-dimethylthiazol-2-yl)-2,5-diphenyltetrazolium bromide (MTT) solution 100 μL of 5 mg/mL was added to each well and incubated in a dark environment at 37 °C for 4 h. After the incubation, MTT solution was replaced with 100 μL DMSO to dissolve formazan product in the 96-wells at 37 °C for 30 min. The formazan crystals were read using a spectrophotometer (Biotrak II Visible Plate reader, Amersham Biosciences Co., Piscataway, NJ, USA) at an absorbance of 570 nm. Five replicas for extract and control were made. The experiments were carried out three times. The living cells (% viability) were calculated using the average optical density (OD) of the experimental groups and divided by the average OD of control wells.

### 3.3 Skin irritation test in the rabbit

**Fig. 1:**
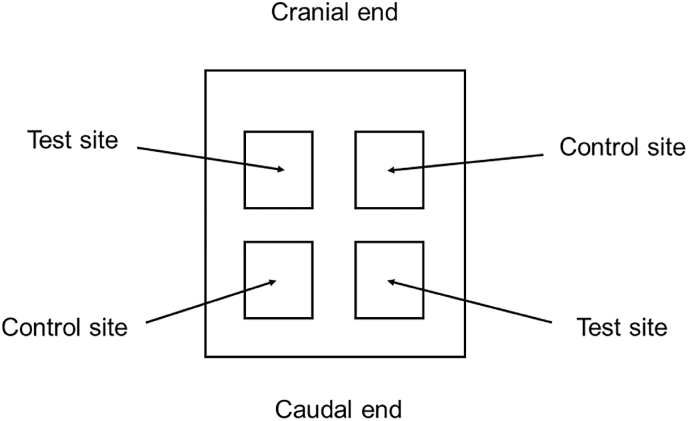
Location of skin application sites in skin irritation test (rabbit).

The adhesive was tested in a rabbit dermal irritation. This study was approved by the Thailand Institute of Scientific and Technology Research (TISTR) with the Animal Ethics Committee (approval number TS-62031) and conducted according to the ISO 10993 part 10.^15^ Three healthy adult albino rabbits of New Zealand white hybrid strain, weighing not less than 2 kg was used. One day before experimentation, rabbits were clipped free of fur on the back region of 10 × 15 cm^2^, and two areas of the shaven skin of approx. 2.5 × 2.5 cm^2^ were selected. Then 0.5 mL of the adhesive was introduced onto a 2.5 × 2.5 cm^2^ gauze patch, and 0.5 mL of distilled water was also introduced on another patch. The patches were applied to each skin site as shown in Fig. 1. The application sites were covered with transparent film dressing and non-irritant adherent tape for 4 h. Then, they were removed, and the positions of each site were marked with permanent ink. The residual test materials were removed using lukewarm water and dried carefully.

### 3.4 Observation and evaluation of animals

All animals were observed daily for clinical signs. Natural lighting was used for skin reaction observation. A scoring system for skin reaction was used to describe the skin reaction for erythema and edema (Table 3).

**Table 3:** A scoring system for skin reaction was used to describe the skin reaction for erythema and edema according to ISO 10993 part 10.^15^

Score	Response category
Erythema and eschar formation	
0	no erythema
1	very slight erythema
2	well-defined erythema
3	moderate to severe erythema
4	severe erythema and slight eschar formation
Edema formation	
0	no edema
1	very slight edema
2	slight edema
3	moderate edema
4	severe edema

Each application site appearance was recorded at 24 ± 2 h, 48 ± 2 h, and 72 ± 2 h following the removal of the patches. For any response, the maximum irritation score was recorded as the maximum primary irritation index (PII), time of onset of response, and time to maximum response. The cumulative irritation index was characterized by the score and description of the response category (Table 4). All rabbits were euthanized by intravenous injection of thiopental after experimental termination.

**Table 4:** Primary or cumulative irritation index categories in a rabbit according to ISO 10993 part 10.^15^

Mean score	Response category
0–0.4	Negligible
0.5–1.9	Slight
2.0–4.9	Moderate
5.0–8.0	Severe

### 3.5 Skin irritation test in healthy volunteer

**Fig. 2:**
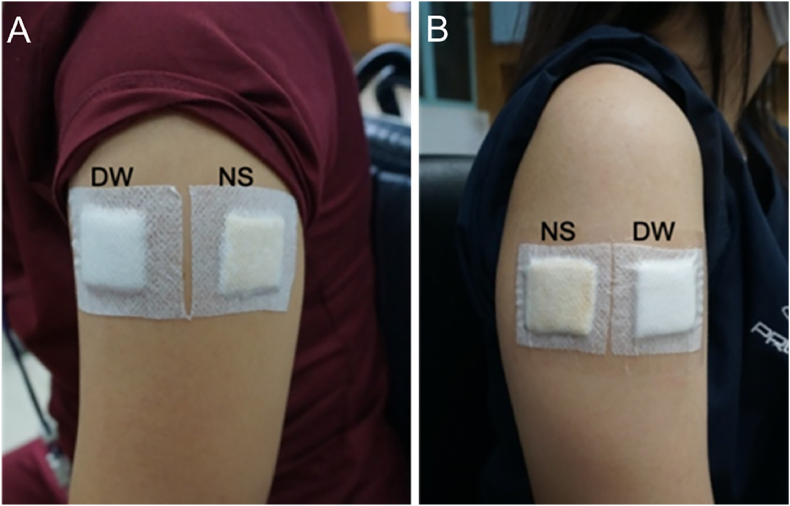
Skin irritation test in humans. Location of the samples treated with deionized water (DW) and new DNRL skin adhesive (NS) at 4 h closed-patch exposure (A) and 24 h closed-patch exposure (B).

The new DNRL skin adhesive and control (distilled water) were studied for dermal irritation in healthy volunteers. This study was approved by The Research Ethics Committee (REC) (approval number EC6202-03-P-HR) and conducted according to ISO 14155-1.^16^ The research was conducted on forty healthy volunteers (n = 40), 18–60 years of age with no sign of dermatitis, not pregnant, and not breastfeeding. Information protocols were given to the volunteer and the informed consent statement was obtained before the study. The volunteers complied with exercise restrictions during the test period to keep the test area dry until the last reading as exercise can cause sweating. The skin irritation was performed on the upper arm. A cotton pad size 2.5 × 2.5 cm^2^ was placed on each stainless tray and saturated with 2 mL of the test material to cover the surface of the pad and apply the test material to the upper outer arm (Fig. 2). The distilled water was also introduced on another pad. The application sites were cleaned with 70% ethyl alcohol until the skin was clean and oil-free to standardize skin condition before the tests. The samples were secured with non-allergic porous, transpore tape (Micropore, 3 M). The test materials were occluded on the upper outer arms in 2 sets. The first set was occluded for 4 h. If there are no signs of irritation after being elevated for at least 48 h, the second set was occluded for an additional 24 h on another upper outer arm. After that, the patches were removed and immediately observed for any signs of skin irritation at 1, 2, 24, 48, and 72 h after patch removal. The sites were evaluated before the initial application of the test material and after the time periods described above. The same assessor assessed the test site reactions for irritation in increasing severity using a grading scale from grade G0 to G3, as shown in Table 5. Photographs of the skin reaction were taken using a digital camera. Any volunteer with severe irritation was excluded from further evaluation.

**Table 5:** Human skin irritation test, grading scale according to ISO 14155-1.^16^

Grading	Description of response
G0	No reaction
G1	Weakly positive reaction (usually characterized by mild erythema and/or dryness across most of the treatment site)
G2	Moderately positive reaction (usually distinct erythema or dryness, possibly spreading beyond the treatment site)
G3	Strongly positive reaction (strong and often spreading erythema with edema and/or eschar formation)

### 3.6 90-degree peel test

**Fig. 3:**
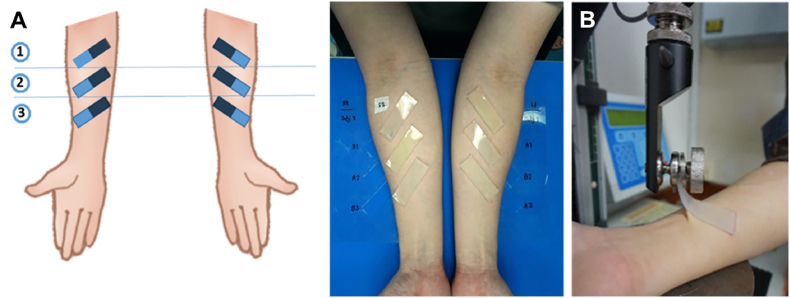
Skin tests showing no irritating reaction in humans. Before (A), During (B) and After treatment (C).

The new DNRL skin adhesive and control were studied for 90-degree peel bond strength and evaluated the effectiveness of the adhesive to skin and adhesive failure. From the pilot study, the average and variance of commercial and DNRL adhesives were estimated for the sample size calculation. The sample size was calculated from the G*power program involving an independent T-test. This analysis showed that a minimum of 13 human subjects would be needed for the study but we chose 20 subjects to provide adequate statistical power to detect a difference in the bond strength between adhesives. The volunteers without signs of skin irritation who passed the skin irritation test were allocated to this test using a simple random sampling method and assigned the volunteer code. Skin preparation was done for the subjects; forearms were washed with soap and water and cleaned with 70% ethyl alcohol to standardize skin condition before the tests. The specimens were fabricated with MDX4-4210® (base/catalyst ratio 10:1) according to the manufacturer's instructions into 60 x 20 × 3 mm^3^ by using gypsum molds. The presence of many bubbles in silicone strips was excluded. Then a thin layer of adhesive was applied on a silicone strip and weighed before adhering and then allowed to dry for approx. 4 mins. The polyvinyl chloride transparent sheets were used to define all 6 sites of each subject's right and left arms. The location of the test specimen was determined randomly at the three sites located between the wrist and elbow of each arm, angled in the inferiolateral to superiomedial direction toward the subject's head (Fig. 3A).

The single-blind procedure was used to guard against measurement bias. The volunteer code and adhesive code (A and B) for each subject were recorded on the transparent sheets for subsequent repositioning. The specimens were adhered to on the ventral forearm with control and the new DNRL skin adhesive (3 silicone strips per adhesive). During the 4 h test period, the subject covered their arms with long sleeves and did not limit their normal activities to indoors. After the 4 h test period, the silicone strips were peeled off from the skin at a 90° angle using the universal testing machine (Lloyd instruments, LRX-Plus, AMETEK Lloyd Instrument Ltd., Hampshire, UK) at a crosshead speed of 600 mm/min until failure occurred. Peeling is in the inferolateral-to-superomedial direction (Fig. 3B). The removal force was recorded as the maximum force and then divided by the width of the specimen which is the peel bond strength (N/m) for that adhesive formulation.

The secondary outcome was identified and categorized as either the outcome adhesive remained on the strip of prosthetic material, or the adhesive remained on the forearms of the participant. After that, the adhesive was removed from the skin and silicone strips by gently rolling the adhesive off with fingertips and washing it with soap and water to remove any residue. The 70% ethyl alcohol or soap without lotions was recommended to remove the remaining adhesive on the silicone strips.

### 3.7 Satisfaction questionnaire

The volunteers were questioned regarding a score of satisfaction (1–5) of each formulation following assessment topics in odor satisfaction, prosthesis adherent, easily removing prosthesis, removing residual adhesive from the skin, removing residual adhesive from the silicone, and overall satisfaction. The observation form was used along with a questionnaire to observe volunteers. The degree of satisfaction ranged from 1 (absolutely dissatisfied) to 5 (completely satisfied).

### 3.8 Statistical analysis

Descriptive statistics were calculated for all experiments and expressed as mean ± standard deviations. Descriptive statistics were also utilized to describe the rabbit skin irritation test as well as volunteer satisfaction. Data analyses were done using SPSS version 24 (IBM, Corp.; Armonk, NY, USA). The McNemar test was used to evaluate the human skin irritation test. Test for the normality was done using the Shapiro-Wilk test, and homogeneity of variance was carried out using Levene's test for all the measured cell viability testing and 90-degree peel testing. When significant values were found. The cell viability was compared using Two-way ANOVA. Tukey's HSD test was used to evaluate differences between the groups. The peel bond strengths were compared in two groups with independent samples *t*-test at a significance level of *p<*0.05.

## 4 Results

### 4.1 Biocompatibility testing (Cell viability study)

The results of cell viability testing at 24, 48, and 72 h are shown in Table 6. The exposure of the HaCat cell line to the adhesive tested of the new DNRL skin adhesive resulted in the highest percentage of cell viability at all periods. The new DNRL skin adhesive showed significantly higher cell viability than the Daro-Hydrobond adhesive (positive control) at all incubation periods (*p<*0.05). The cell viability of positive control and negative control (pure silicone: MDX4-4210) showed no significant difference in all incubation periods.

**Table 6:** The percentage of cell viability after 24, 48, and 72 h in the adhesive tested groups.

Test materials	Time (h)	N% Grade of skin reaction (n = 40)		Mean ± SD of severity grade	p-value*
		Neg	Pos (G1)		
Distilled water	1	40(100)	–	–	0.50
New DNRL skin adhesive	1	38(95)	2(5)	0.05 ± 0.22	

The different capital letters in the superscripts indicate statistical differences between experimental groups (rows) (*P*<0.05) as the different lower-case letters indicate significant differences between incubation periods (columns) (*P*<0.05).

In the comparison of the different test times, the experimental adhesives in all groups resulted in high cell viability at 24 h incubation period. The cell viability significantly decreased after 24 h of incubations (*p<0*.*05*); however, the decrease was not significant differences from 48 to 72 h on the negative and positive control (*p>0*.*05*). While the cell viability on the new DNRL skin adhesive did not significantly increase after 48 h.

The microscope image of HaCaT cells incubated with medium extract of negative control (Silicone), positive control (Daro adhesive), and the new DNRL skin adhesive showed the extent of cell adhesion on the well plate as non-cytotoxic substrates were similar to the negative control. The images showed a vast amount of cell aggregates in the form of ripple-like areas adhered on the surfaces, with the typical keratinocyte cell shape which revealed normal morphology and proliferation pattern.

### 4.2 Skin irritation in the rabbit

According to the results, the mean of PII for both distilled water and Daro-Hydrobond adhesive was less than 0.5, indicating that neither caused significant skin irritation, whereas the new DNRL skin adhesive caused moderate irritation. The mean PII of each rabbit of the new DNRL skin adhesive was 4.8, 3.5, and 2.8. Under the condition of this study, the new DNRL skin adhesive produced moderate erythema and edema skins, however, the skin lesions recovered within 14 days. The residual new DNRL skin adhesive was strongly attached to rabbit skin and was difficult to remove, which caused erythema and edema. Moreover, the residual Daro-Hydrobond adhesive left hard edges on rabbit skin that persisted until the final day of the 14-day testing period, and around the hard edges, showed mild erythema and edema skins, however, the skin lesions recovered within 7 days.

### 4.3 Skin irritation tests

**Fig. 4:**
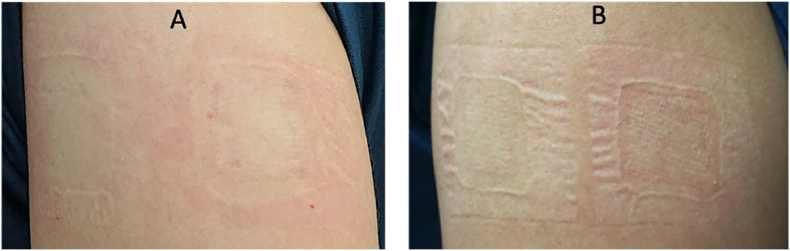
Skin tests showing irritating reaction in humans. The irritating reaction on the upper outer arm of two volunteers (G1 reaction to micropore surgical tape (A) and to the new DNRL skin adhesive (B)) within the first hour of the occlusion period, which subsided within an hour without treatment.

**Fig. 5:**
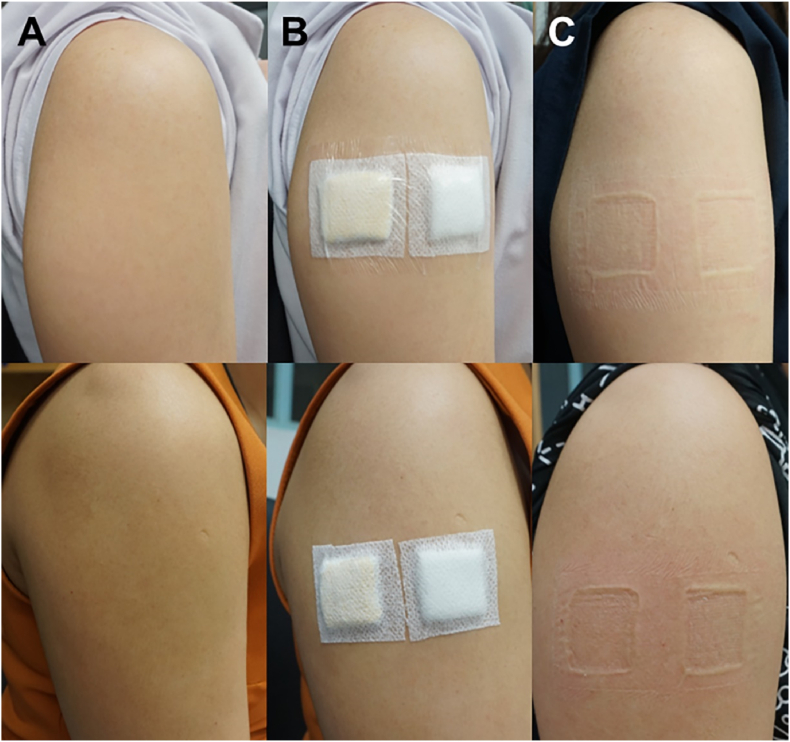
90-degree peel test. Location of skin application sites in 90-degree peel test (A), Peel testing at a 90-degree angle from inferiolateral to superiomedial direction (B).

All 40 volunteers stayed until the study was completed. Two volunteers developed irritation within 1 h occlusion period, one volunteer developed an allergic reaction (grade 1) to the micropore surgical tape on the application sites, and another volunteer developed mild erythema (grade 1) in response to the new DNRL skin adhesive test material, whereas distilled water did not cause an allergic reaction. All these decreased within an hour without any treatment (Fig. 4). The results of the 4 h closed-patch exposure tests are shown in Table 7. These two volunteers were excluded from further evaluation. None of the volunteers developed irritation during or after the 24 h closed-patch exposure (Fig. 5). The skin irritation reactions of the new DNRL skin adhesive were not different from the negative control (*p>*0.05).

**Table 7:** Number of volunteers (percentage) and the grade of skin irritation reactions at 4-h closed-patch exposure.

Test materials	Mean ± SD (n = 20)	p-value	Failure (ss/si)
Daro-Hydrobond adhesive	131.52 ± 21.72	0.434	2/18
New DNRL skin adhesive	103.61 ± 23.18		16/4

ss/si = the number of test specimens where failures occurred at the silicone strip (ss) or skin interfaces (si).

### 4.4 90-degree peel tests

The *in vivo* evaluations of 90-degree peel bond strength in human skin are shown in Table 8. The Daro-Hydrobond adhesive's peel bond strength was 131.52 ± 21.72 N/m, while the peel bond strength of the new DNRL skin adhesive was 103.61 ± 23.18 N/m. Two test adhesives were not different (*p>*0.05). The secondary outcome showed that Daro-Hydrobond adhesive remained adhered on the silicone strip 90%, whereas the new DNRL skin adhesive adhered to the skin 80% and was easier to remove from the substrate than commercial adhesive.

**Table 8:** Mean ± SD of 90-degree peel bond strength (N/m) and mean difference of the test adhesives.

Experimental groups	24 h	48 h	72 h
Negative control (Silicone)	115.06 ± 5.54^Ba^	103.88 ± 13.11^Bb^	98.94 ± 7.90^Bb^
Positive control (Daro-Hydrobond adhesive)	122.44 ± 4.69^Ba^	93.52 ± 12.84^Bb^	92.02 ± 13.14^Bb^
New DNRL skin adhesive	138.39 ± 4.05^Aa^	124.06 ± 10.09^Ab^	135.74 ± 14.66^Ab^

The test materials with no skin reactions at 24 h closed-patch exposure are not shown.

*McNemar test.

### 4.5 Satisfaction

It showed that the volunteers’ satisfaction with the new DNRL skin adhesive ranged from slightly satisfied to completely satisfied, proving satisfaction of more than 90% for all topics. In addition, Daro-Hydrobond adhesive ranged from slightly satisfied to completely satisfied, exhibiting satisfaction levels of more than 75% for almost all topics, whereas the percentages of volunteers who were slightly dissatisfied and neither dissatisfied nor satisfied with the removal residual adhesive from the silicone were approximately 30% and 70%, respectively. In addition, there were no reports of skin irritation or allergic reactions among the volunteers during the application period. The mean of volunteer satisfaction with the test adhesives for all topics.

## 5 Discussion

In this study, the viability of *in vitro* keratinocytes was used to evaluate the biocompatibility of adhesives. Using rabbit and human skin irritation, a 90-degree peel, and satisfaction surveys, the performance and safety of the new DNRL skin adhesive for human skin application were assessed and compared to a commercial adhesive.

The new DNRL skin adhesive was a blend of DNRL, adhesive polymer, and tackifiers that provided the adhesive property of DNRL for adhering silicone maxillofacial prostheses to human skin without creating any adverse effects. The adhesive was biocompatible for keratinocytes’ cell viability. Very mild skin irritation was observed in both rabbits and humans, which appeared as a mild rash, and then rapid recovery of the skin lesions. Twenty volunteers' responses varied from “slightly satisfied” to “completely satisfied” across all questionnaires. The DNRL was safe to apply to the skin as the allergic proteins in the serum of the NRL were eliminated before preparing the adhesive.^9^^,^^17^ There are very few reports of DNRL being used in medical applications such as transdermal drug delivery, cosmetic pore packs, and peel-off masks. Previous research showed that 20% PVA at 15 phr was an appropriate polymer for forming DNRL adhesive with proper adhesive properties, and had adequate peel bond adhesive strength similar to the commercial adhesive and the new DNRL adhesives were also physically stable after being stored at 4 °C.^13^

The biocompatibility of the novel adhesive can be evaluated in a variety of ways. To investigate the potential cytotoxic effects of the adhesives on the HaCaT cell line, the MTT assay is a good indicator of cell viability.^18^ MTT is a sensitive index for determining the cytotoxicity of adhesives.^19^ In this study, HaCaT cells were exposed to different adhesives from 24 to 72 h because cell survival tended to increase over time. The duration of cell/adhesive exposure in the test system should consider *in vivo* lifetime. Cytotoxicity reactions under clinical conditions might be different than those observed in cell culture tests. Thus, the MTT assay was only used for toxicity screening. The HaCaT cells could continuously grow or proliferate on 24-h incubation period for all experimental adhesives. From these results, it could be indicated that the adhesives extracted by the culture medium did not inhibit cell proliferation. Especially, the cell proliferation under the new DNRL adhesive medium was higher than under other media followed by positive control and negative control. It was worth mentioning that for the studied sample preparation. The new DNRL adhesive has a pH of approx. 7, which corresponds to the pH required by the HaCaT cell, and the control of pH is important for optimal culture.^20^ In recent study showed that the biocompatibility of DNRL to fibroblast cells (L-929) is a strong strategy for developing biocompatible NRL.^21^ The data from the 48-h incubation period indicated that the long incubation time resulted in a significant decrease in viable cells because of nutrient depletion which was related to improper balance between the supplement in the extracted medium and the number of cells.^22^ Moreover, these results could be a confounding factor in the analysis of cytotoxicity results, as is improper of insoluble formazan product which MTT assay require DMSO for dissolution insoluble formazan product. After the 48-h incubation period, the cell that grew in the extracted medium of the new DNRL adhesive could continuously proliferate. This information is similar to the cytotoxicity assay results with high standard deviation on 48- and 72-h incubation periods and the difference in cell viability rates amongst the experimental adhesives could be related to the variation in their chemical composition and amount of chemotoxic leachable moving from these adhesives. From the results, it could be assumed that the new DNRL adhesive could be considered to be safe and subsequently tested for skin irritation on animals.

The new DNRL skin adhesive was tested in acute dermal irritation which produced moderate erythema and edema skin. These symptoms were detected during wiping with moistened cotton wool because the adhesive had attached to rabbit fur and was hard to remove. Therefore, the removal technique should be gently wiped and avoided rub to remove any residual test adhesive. The mean PII of each rabbit was 4.8, 3.5, and 2.8 which indicated that the variation could be caused by the cleansing technique. Also, the variability was usually found in skin irritation tests.^23^ However, it subsided within 14 days in all three rabbits. The new DNRL skin adhesive can be indicated as safe for various skin applications but careful in people who have sensitive skin. These findings coincided with the results of previous studies that found the safety of the DNRL, the peel-off masks, and cosmetic pore packs prepared from DNRL, and this irritation could be gone without treatment.^11^^,^^12^^,^^24^ In addition, the remaining Daro-Hydrobond adhesive left hard edges on rabbit skin that remained until the last day of the 14-day testing period; the area around the edges exhibited mild erythema and edema skins, but the lesions healed after 7 days.

The human closed patch test was done to assess the skin irritation of the new DNRL skin adhesive. The readings on skin irritation reactions were done carefully. One subject showed an allergic reaction to the micropore surgical tape surrounding the patched region, probably because of skin occlusion-induced irritation or the presence of moisture. This is consistent with reports of surgical tape allergies associated with long-term use, and removal of the tape produces irritation in patients with fine hair. Another subject had a positive response to the new DNRL skin adhesive decreased within 1 h without any treatment. The results revealed that the adhesive could induce mild irritation and inflammation on the skin. DNRL can be included in peel-off masks in paste form which showed a mild level of skin irritation in the rabbit, but this irritation can subside without treatment while healthy volunteers showed high satisfaction with no evidence of skin irritation.^11^ There was no skin irritation in the rabbits, and they were safe for skin application.^12^ When evaluating the overall irritation reaction, the result showed that the new DNRL skin adhesive was not different from the negative control. In the current study, the first group was occluded for 4 h without showing any signs of irritation. The second group was then occluded for 24 h to evaluate long-term irritation. The results indicate that none of the volunteers experienced any irritation during the 24 h observation period. Furthermore, there was no observed tissue reaction during an extended follow-up period, which is consistent with the fact that patients often wear maxillofacial prostheses for 6–8 h each day. It could be assumed that the new DNRL skin adhesive could be considered safe for skin application.

The peel test was used to determine the most suitable adhesive formulations because it is more predictive of a material's clinical adhesion and stimulates the horizontal component of the peeling force created when the patient pulls the prosthesis from the defect site.^25^ Polyvinyl chloride transparent sheets were used to identify all six sites on the volar surfaces of each subject's right and left arms to control the position and peel angulation in the inferiolateral to superiomedial direction. To ensure an even distribution of adhesive type throughout all testing sites, the test specimen was placed in a stratified random pattern between the three sites located between the wrist and elbow of each arm. The volunteers were billed by type of adhesive during the test to prevent measuring bias. Although the Daro-hydrobond adhesive (131.52 ± 21.72 N/m) had a higher peel bond strength compared to the new DNRL skin adhesive (103.61 ± 23.18 N/m), there was no significant difference between the adhesives. The relative skin bond strengths of Secure medical adhesive (a silicone-based adhesive) to silicone materials (91.1 ± 36.30 N/m) were the same as the previous study, which was evaluated at 4 h.^26^ The new DNRL skin adhesive with the proper adhesive properties was formed by blending 20% PVA at 15 phr with DNRL. PVA's remarkable properties are related to the molecule's structure, which contains numerous hydroxyl groups that could create hydrogen bonds.^27^ Previous research found that combining PVA and DNRL could improve the adhesiveness of DNRL for pharmaceutical applications such as the nicotine transdermal patch.^9^^,^^10^ By incorporating tackifier resin into DNRL, adhesion was enhanced by the tackifier resin, which improved substrate wettability and improved bonding.^28^ Moreover, 80% of the Daro-Hydrobond adhesive residue remained on the silicone strip, while 82% of the new DNRL skin adhesive residue remained on the skin and was easier to remove than commercial adhesive. The debonding occurred at the skin interface for Daro-Hydrobond adhesive, as residual remained on silicone material as in the previous study.^8^

The volunteers' satisfaction was generally satisfied with both adhesives after a peel test, with high degrees of satisfaction among all topics. However, those who used the Daro-Hydrobond adhesive reported being slightly dissatisfied with the removal of residual adhesive from the silicone, presumably because more adhesive remained on the strip after removal. Although the volunteers were instructed to utilize the removal method, Daro-Hydrobond adhesive still has been presented.

There are certain limitations of this research. No microbial analyses were done to confirm microorganisms in the adhesive as the microorganisms can play a role in the increase in sensitivity of adhesive. The most of participants spent indoors during the 4-h study period. The adhesive bond strength results may be different if the subjects spent time outdoors. When subjected to various conditions, such as temperature, humidity, or activity levels, the two adhesives may exhibit different behaviors. Finally, the skin tone and amount of skin oils were not considered in this study and may reflect variations in the patient population. The generalizability of results from the new DNRL skin adhesive to other populations depends on various factors, including skin variability, cultural practices, and environmental factors. These factors can vary widely between regions and ethnicities, which has the potential to impact the effectiveness and durability of the adhesive. Therefore, the adhesive can be used carefully in American or European populations, apart from Asian populations. However, it remains essential to continually monitor patient experiences and gather feedback to ensure that the new DNRL skin adhesive maintains its effectiveness and comfort for patients in their daily use.

## 6 Conclusion

The new DNRL skin adhesive showed comparable peel bond strength and patient satisfaction to those of commercial adhesives. The DNRL skin adhesive was biocompatible for keratinocyte cell viability but resulted in self-resolving mild irritation in animals and humans. Thus, the new adhesive can be used to adhere to the maxillofacial silicone prostheses with some precaution.

## Funding

This research was supported by Research Center of Ecellence for Oral Health, Faculty of Dentistry, Prince of Songkla University, Thailand.
